# Willingness to Use the Internet to Seek Information on HIV Prevention and Care among Men Who Have Sex with Men in Ho Chi Minh City, Vietnam

**DOI:** 10.1371/journal.pone.0071471

**Published:** 2013-08-19

**Authors:** Pauline Justumus, Donn Colby, Thi Mai Doan Anh, Eric Balestre, Renaud Becquet, Joanna Orne-Gliemann

**Affiliations:** 1 Harvard Medical School AIDS Initiative in Vietnam, Ho Chi Minh City, Vietnam; 2 University Bordeaux, ISPED, Centre Inserm U897- Epidémiologie-Biostatistique, Bordeaux, France; 3 Institut National de la Santé et de la Recherche Médicale, ISPED, Centre Inserm U897- Epidémiologie-Biostatistique, Bordeaux, France; 4 Beth Israel Deaconess Medical Center, Boston, Massachusetts, United States of America; Rollins School of Public Health, Emory University, United States of America

## Abstract

**Background:**

In Vietnam, men who have sex with men (MSM) are highly affected by HIV and need new targeted HIV prevention strategies.

**Objectives:**

To assess the willingness to use the Internet to seek information on HIV prevention and care and associated factors among MSM in Ho Chi Minh City.

**Methods:**

A descriptive cross-sectional study was conducted in 2012. Participants were recruited using a convenience sampling method in venues most frequented by MSM and completed a self-administered questionnaire. Logistic regression models were performed to estimate factors associated with the willingness to use the Internet to seek information on HIV prevention and care.

**Results:**

A total of 358 MSM were approached for the survey and 222 questionnaires (62.0%) were eligible for analyses. Overall, 76.1% of the respondents reported that they were willing to use the Internet to seek information on HIV prevention and care. A number of male partners in last year less than or equal to 3 (Adjusted Odds Ratio: 3.07, 95% Confidence interval: 1.40–6.73), a history of STI screening (4.10, 1.02–16.48) and HIV testing (3.23, 1.20–8.64) and having ever sought a male sexual partner through the Internet (3.56, 1.55–8.18) were significantly positively associated with the willingness to use the Internet to seek information on HIV prevention and care.

**Conclusion:**

The MSM interviewed in Ho Chi Minh City reported a high willingness to use the Internet to seek information on HIV prevention and care. In a context where new media are increasingly considered as promising options for reaching this HIV risk group, further research should be conducted on developing and testing tailored online tools adapted to the needs of Vietnamese MSM.

## Introduction

Since the first cases of AIDS reported in the early 1980s in the gay community of San Francisco, HIV prevalence has been consistently higher among men who have sex with men (MSM) than in the general population and MSM remain at high risk for HIV infection worldwide [1], [2], [3]. The MSM population has remained largely understudied, under-informed and not well targeted by public health policies and programs in many countries [4]. In 2012, the emergence or re-emergence of an HIV epidemic among MSM was shown in various settings [5], [6], [7].

In Vietnam, with an estimated 280,000 HIV-infected people, the HIV epidemic is still at a concentrated stage and MSM are one of the groups with the highest HIV prevalence [8]. Although socioeconomic development has changed attitudes towards MSM in the last 10 years, homosexuality is generally not considered as a normal or acceptable practice in Vietnam [9], [10]. Men who engage in homosexual practices tend to be regarded as a “deviant” group, and therefore rarely disclose their sexual orientation [11].

It has been estimated that the proportion of MSM living in the two largest cities of Vietnam, Ho Chi Minh City and Hanoi (2%), was higher than in the rest of the country (1%) [12]. MSM indeed tend to be attracted by large urban settings where they can more easily socialize, access HIV prevention services, and openly discuss health issues related to their sexual orientation [13]. HIV infection rates have recently increased among MSM, from an estimated 9.0% in 2006 to 16.7% in 2010, while HIV prevalence in the general adult population has remained stable, around 0.4% [8], [14], [15]. In Ho Chi Minh City over the 2009–2011 period, the HIV prevalence estimated among MSM ranged between 14.3 and 16.3% [16], [17].

This high HIV burden among MSM in Vietnam has been explained by individual risk-taking such as unprotected anal intercourse, multiple sex partners, history of drug use and history of sexually transmitted infections (STI) [18], [19], [20]. Moreover, as MSM in Vietnam experience stigma and discrimination, they face many barriers to accessing health care and HIV prevention messages [9], [21]. The inadequate access to information contributes to low levels of HIV knowledge among MSM, which in turns contributes to high-risk behaviors and elevated HIV prevalence [15]. Further, as in many other countries in the region, very few HIV programs for MSM have been implemented in Vietnam, partly due to social resistance and lack of political will [4].

However, the Vietnamese government has recently demonstrated increasing interest in prevention and care interventions targeted for MSM. The country is indeed currently working towards strategizing how to implement specific HIV services for MSM in the context of decreasing international donor funding. Yet promising prevention strategies that lower biological transmission, such as antiretroviral-based interventions [22], are constrained by the low health-seeking behaviors of MSM populations [23]. Hence, new targeted HIV prevention and care strategies, that are cost-effective and acceptable by the majority of MSM, now need to be defined and implemented in Vietnam.

New media offer opportunities for large-scale interventions in this context [24]. The Internet is a means for networking and seeking sexual partners among MSM [25]. It has recently emerged as a tool for health promotion on HIV prevention and is already used in some countries for online HIV and STI testing [26].

Internet-based interventions targeting individual sexual risks enhance the effectiveness of barrier (condom) and biomedical (pre-exposure treatment with antiretrovirals) prevention strategies and facilitate linkage to care [24]. Internet-delivered HIV prevention interventions, consisting of interactive activities and conversation with medical experts, contributed to a significant increase in HIV/AIDS-related knowledge and safer sex attitudes among rural MSM in the United States [27]. In another rural population, Internet-based prevention modules contributed to reduce the number of sexual partners and increase condom use [28]. A 90-min Internet-based safer sex intervention led to a reduction in unprotected sex practices among American MSM [29]. A randomized trial in the United States recently showed that an Internet-based sexual health promotion intervention reduced short-term unprotected anal intercourse among MSM [30]. Finally, an intervention designed to promote HIV testing among MSM within existing Internet chat rooms [31] showed increased self-reported HIV testing among chatters.

These different Internet-based interventions have many advantages: guaranteed anonymity, low cost, and potentially high accessibility. They can increase the delivery of HIV prevention messages to a diverse at-risk audience, reducing barriers due to stigma and discrimination. Moreover, they can reach a relatively large number of individuals from the target population in a short period of time [32]. To date, Internet use for HIV prevention and care has been mostly studied in high-income settings, such as in the United States or in Canada. However, it is now being considered in resource-constrained settings, following the increased coverage of the Internet worldwide, regardless of a country's level of development [33]. The use of Internet-based information strategies could represent for many countries a cost-effective way to improve HIV awareness and knowledge of MSM who might be missed by current interventions such as peer education or general mass media campaigns [34].

Over the last few years, Vietnam has been experiencing a more rapid growth of Internet use than any other country in the region. In 2010, it was estimated more than 50% of the urban population had ever used the Internet, this proportion being even higher among young people [35]. A cross-sectional study among MSM in rural and urban areas of the Khanh Hoa Province showed that 30% of MSM interviewed often used the Internet to meet other MSM [36]. Several gay websites already exist in Vietnam and are commonly used by MSM. In this context, Internet could therefore potentially be an interesting tool to improve HIV knowledge and awareness among MSM. However, the willingness to use the Internet to be informed on HIV prevention and care among Vietnamese MSM has not yet been studied.

Our objective was to investigate the willingness to use the Internet to seek information on HIV prevention and care among MSM in Vietnam and its associated factors.

## Methods

### Study design and study sites

A cross-sectional study was carried out among MSM in Ho Chi Minh City in September 2012. We identified the most frequented public parks, cafés, bars and saunas for MSM. A first site was identified by the local partners and the snowball sampling method was then used among the personal working at this facility to identify other sites potentially eligible for the study [37]. Authorization from the manager of each of these private entertainment establishments was sought.

### Participants and recruitment

MSM were recruited during selected recruitment periods, to fit with the days and times when they frequented the study sites. At each study site, MSM were recruited using a convenience sampling method. Men were approached, briefly explained the scope of the study and assessed for eligibility. Inclusion criteria were age ≥18, reporting having had sex with a man in the past year and being Vietnamese citizen. After a detailed description of the study and its potential risks and benefits, oral informed consent was obtained from each participant prior to the initiation of any study procedures. An identification number was allocated to each participant.

### Sample size

Sample size calculation was based on an expected percentage of MSM having ever used the Internet to access information about HIV prevention and care of 30%, with an expected confidence interval precision of ±6% [36]. The minimum sample size required was 224 MSM, taking into account a refusal rate of 20% and an expected proportion of non-analyzable data of 15%. We aimed at identifying a total of 308 MSM over the study period.

### Data collection

Participants were given a self-administered questionnaire to be completed in Vietnamese, documenting their socio-demographic characteristics, HIV knowledge, HIV risk behaviors and willingness to use the Internet to seek information on HIV prevention and care. Knowledge of HIV/AIDS was assessed using a mean score developed from seven questions regarding HIV transmission and concepts; this score was created specifically for the study, based on different score constructions derived from the literature [38], [39]. The list of different Internet tools providing information, listed in Table 1, was defined based on previous studies documenting Internet use [40], [41], [42].

**Table 1:** Willingness to use the Internet and types of Internet tools to seek information on HIV prevention and care of men who have sex with men, 2012, Vietnam.

Characteristics		Number (%) (n = 222)
***Ever used the Internet to seek information on HIV prevention and care***	Yes	136 (61.3)
	No	86 (38.7)
***Willing to use the Internet to seek information on HIV prevention and care***	Yes	169 (76.1)
	No	53 (23.9)
***Type of Internet tools to seek information on HIV prevention*** *	Websites	163 (91.4)
	Missing	6
	Social Network	120 (73.2)
	Missing	5
	Personal email box	104 (64.2)
	Missing	7
	Chat room	101 (62.7)
	Missing	8
***Type of Internet tools to seek information on HIV care*** *	Websites	146 (89.0)
	Missing	5
	Website with HIV-infected people testimonies	123 (75.0)
	Missing	5
	Social Network	111 (68.9)
	Missing	8
	Chat room	110 (67.9)
	Missing	7
	Personal email box	106 (65.4)
	Missing	7

* Regarding potential Internet tools, question was: To seek information on HIV prevention/care, would you use.

-“A website where you can find what you need?”

- “A chat room where you can have instant message with a health worker?”

- “Your email box where you can receive HIV prevention/care information?”

- “Social networks like Facebook, Twitter, Pinterest, Foursquare, Tumblr?”

- “A website where you can read stories about people living with HIV?”

### Statistical methods

The main study outcome was the willingness to use the Internet to seek information on HIV prevention and care, defined as MSM responding yes to the following question: “Would you like to use the Internet to have information on HIV prevention and care?” Each characteristic of MSM was categorized in binary variable and was described according to the willingness status of MSM.

To calculate the HIV knowledge score, one point was given for each correct answer and no point for each incorrect or unknown answer. “High knowledge” was defined as an HIV knowledge score above the median score.

To define the socio-behavioral factors independently associated with the willingness to use the Internet to seek information on HIV prevention and care, we first conducted univariable logistic regressions. Variables found to be statistically associated with a p-value <0.2 were included in the multivariable model. To select the final adjusted model, we used a backward elimination method using a p-value of 0.05 and we controlled for potential confounding factors.

Data were entered in a Microsoft Access database and were analyzed using EpiInfo Version 3.5.1.

### Ethics statement

The study was approved by the IRB at Beth Israel Deaconess Medical Center in Boston, USA (protocol number: 2012-P-000199/1) and by the Hanoi School of Public Health in Vietnam. Oral informed consent was obtained from all respondents.

## Results

### Enrolment

Out of the eight potential interviewing sites identified, data collection took place in two public parks and in one café, at night (9 to 11pm), from Friday to Sunday in September 2012. Agreement from managers to host data collection was not granted in the five other sites.

**Figure 1:**
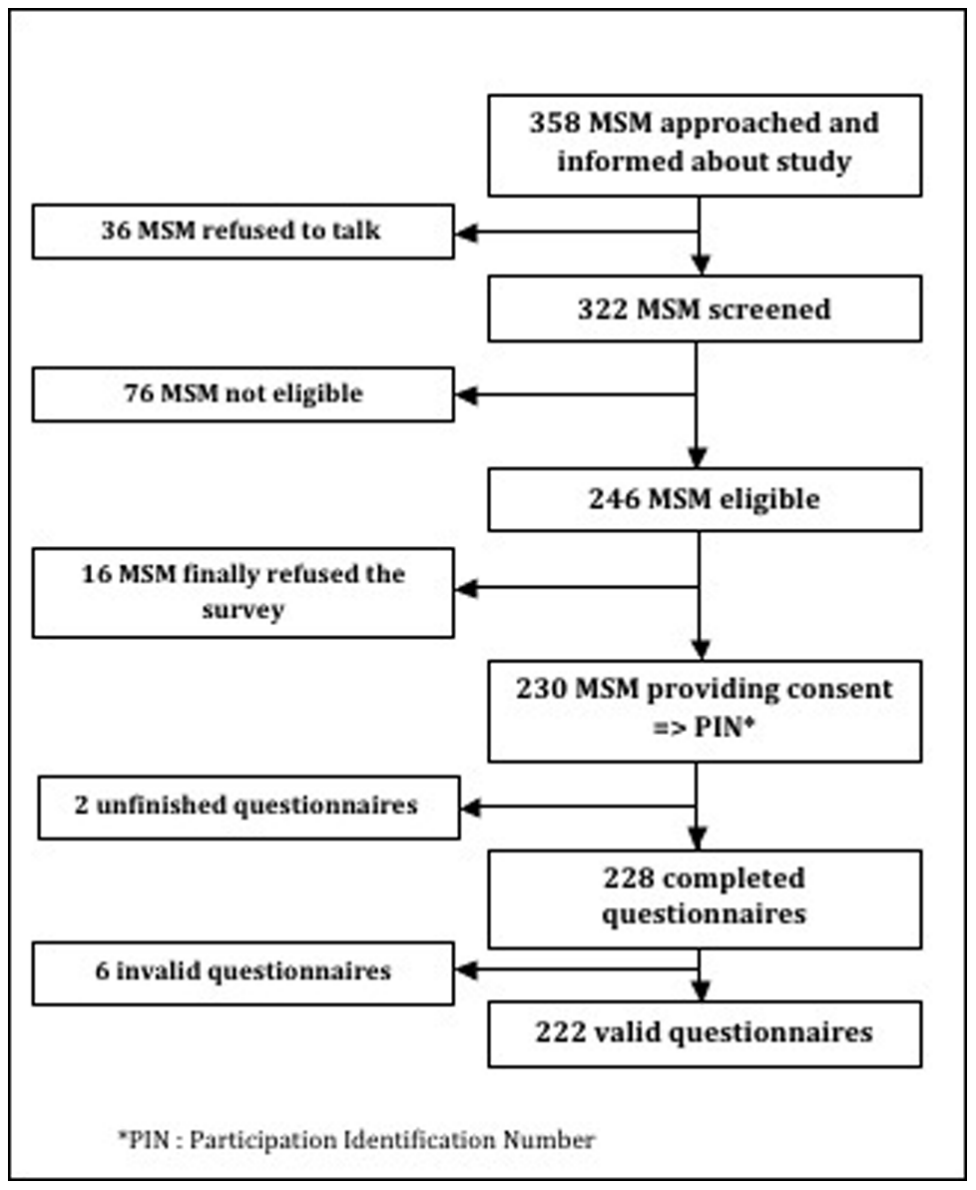
Flow chart of data collection among men who have sex with men.

A total of 358 MSM were approached, 128 either refused to participate in the survey (n = 52, 40.6%) or were not eligible (n = 76, 59.4%), and 230 were enrolled in the study (Figure 1). Eight MSM were excluded during analyses: two provided incomplete questionnaires and six provided invalid questionnaires because they had not actually had sex with a man in the past 12 months (n = 2) or provided inconsistent data (n = 4). Finally, 222 MSM were included in the main outcome analysis, of which 203 were included in the regression analysis of factors associated with the willingness to use the Internet to seek information on HIV prevention and care. The two populations, included in (n = 203) and excluded from regression analysis (n = 19), were overall comparable except for two variables: MSM excluded from the analysis of associated factors were less likely to report consistent lubricant use and to feel at no risk for HIV than those included in the analysis (p-values <0.05).

### Internet use to be informed on HIV prevention and care

Overall, 76.1% of the MSM interviewed were willing to use the Internet to seek information on HIV prevention and care (Table 1). 61.3% had already used it for this purpose.

When asked about which Internet tool they would use to be informed on HIV prevention, 91.4% of MSM indicated that they would use a website with HIV/AIDS information and 73.2% would use social networks. For HIV care information, 89.0% of respondents were willing to use a website with HIV/AIDS information and 75.0% of them a website compiling HIV-infected people testimonies. Chat rooms with trained health care workers and personal email box were less appreciated by the respondents (respectively 62.7% and 64.2% for HIV prevention, 67.9% and 65.4% for HIV care).

### Sociodemographics

Overall, men were young, median age in years was 22 [Inter-quartile range (IQR): 20–26]. Respondents were mostly from the predominant Kinh ethnic group and had completed more than 12 years of education (Table 2). MSM willing to use the Internet to seek information on HIV prevention and care were overall comparable to MSM not willing to use the Internet for such purposes, except for being Kinh, independently associated with the willingness to use the Internet to seek information on HIV prevention and care.

**Table 2:** Factors associated with willingness to use the Internet to seek information on HIV prevention and care among men who have sex with men, 2012, Vietnam.

		Total MSM n = 203	MSM willing to use the Internet for HIV n = 154	MSM willing not to use the Internet for HIV n = 49	Univariable logistic regression		Multivariable logistic regression Final model	
Factors					OR (95% CI)	p-value	aOR (95% CI)	p-value
**Age**	>22 years	98 (48.3)	73 (47.4)	25 (51.0)	1.00			
	≤22 years	105 (51.7)	81 (52.6)	24 (49.0)	1.15 (0.61, 2.20)	0.66		
**Ethnicity**	Kinh	189 (93.1)	147 (95.5)	42 (85.7)	1.00			
	Hoa (Chinese)	14 (6.9)	7 (4.5)	7 (14.3)	0.85 (0.74, 0.98)	0.02		
**Educational level**	More than high school	152 (74.9)	116 (75.3)	36 (73.5)	1.00			
	High school or less	51 (25.1)	38 (24.7)	13 (26.5)	0.90 (0.43, 1.88)	0.79		
**Sexual orientation**	Homosexual	172 (84.7)	130 (84.4)	42 (85.7)	1.00			
	Others	31 (15.3)	24 (15.6)	7 (14.3)	1.11 (0.44, 2.77)	0.82		
**Age at first sex with male partner**	>18 years	100 (49.3)	76 (49.4)	24 (49.0)	1.00			
	≤18 years	103 (50.7)	78 (50.6)	25 (51.0)	0.98 (0.51, 1.87)	0.96		
**Number of male partners in last year**	>3	97 (47.8)	67 (43.5)	30 (61.2)	1.00		1.00	
	≤3	106 (52.2)	87 (56.5)	19 (38.8)	2.05 (1.06, 3.95)	0.03	3.07 (1.40, 6.73)	0.005
**Sexual intercourse with a female in last year**	No	182 (89.7)	138 (89.6)	44 (89.8)	1.00			
	Yes	21 (10.3)	16 (10.4)	5 (10.2)	1.02 (0.35, 2.94)	0.97		
**HIV Knowledge high score** * **(>5/7)**	No	121 (59.6)	88 (57.1)	33 (67.3)	1.00			
	Yes	82 (40.4)	66 (42.9)	16 (32.7)	1.50 (0.78, 2.88)	0.20		
**Perception of no HIV risks**	No	118 (58.1)	90 (58.4)	28 (57.1)	1.00			
	Yes	85 (41.9)	64 (41.6)	21 (42.9)	0.94 (0.49, 1.81)	0.87		
**Feeling sufficiently or largely informed about HIV**	No	14 (6.9)	12 (7.8)	2 (4.1)	1.00			
	Yes	189 (93.1)	142 (92.2)	47 (95.9)	0.50 (0.10, 2.33)	0.38		
**Consistent condom use with regular male partner in last month**	No	140 (69.0)	107 (69.5)	33 (67.3)	1.00			
	Yes	63 (31.0)	47 (30.5)	16 (32.7)	0.90 (0.45, 1.80)	0.77		
**Consistent condom use with casual male partner in last month**	No	130 (64.0)	98 (63.6)	32 (65.3)	1.00			
	Yes	73 (36.0)	56 (36.4)	17 (34.7)	1.07 (0.54, 2.11)	0.83		
**Consistent lubricant use with male partner in last month**	No	127 (62.6)	95 (61.7)	32 (65.3)	1.00			
	Yes	76 (37.4)	59 (38.3)	17 (34.2)	1.16 (0.59, 2.28)	0.64		
**Screening or examination for STI**	No	148 (72.9)	102 (66.2)	46 (93.9)	1.00		1.00	
	Yes	55 (27.1)	52 (33.8)	3 (6.1)	7.81 (2.32, 26.33)	<0.01	4.10 (1.02, 16.48)	0.04
**HIV test ever**	No	115 (56.7)	73 (47.4)	42 (85.7)	1.00		1.00	
	Yes	88 (43.3)	81 (52.6)	7 (14.3)	6.65 (2.81, 15.73)	<0.01	3.23 (1.20, 8.64)	0.02
**HIV test and results**	No	143 (70.4)	100 (64.9)	43 (87.8)	1.00			
	Yes	60 (29.6)	54 (35.1)	6 (12.2)	3.87 (1.54, 9.67)	<0.01		
**Ever felt discriminated against in health services**	No	117 (57.6)	96 (62.3)	21 (42.9)	1.00			
	Yes	86 (42.4)	58 (37.7)	28 (57.1)	0.45 (0.23, 0.87)	0.01		
**Alcohol use less than or once a week**	No	38 (18.7)	25 (16.2)	13 (26.5)	1.00			
	Yes	165 (81.3)	129 (83.8)	36 (73.5)	1.86 (0.86, 4.00)	0.11		
**Drug use ever**	No	163 (80.3)	125 (81.2)	38 (77.6)	1.00			
	Yes	40 (19.7)	29 (18.8)	11 (22.4)	0.80 (0.36, 1.75)	0.57		
**Seeking male sexual partners via Internet**	No	54 (26.6)	33 (21.4)	21 (42.9)	1.00		1.00	
	Yes	149 (73.4)	121 (78.6)	28 (57.1)	2.75 (1.38, 5.45)	<0.01	3.56 (1.55, 8.18)	0.002

**Univariable and adjusted multivariable logistic regression.**

OR: odds ratio; CI: confidence interval; aOR: adjusted odds ratio.

* The HIV knowledge questions were: 1. A person HIV infected can look healthy outside. 2. HIV can be transmitted by eating a meal with an HIV infected person. 3. A person can get HIV by having anal sex with an HIV infected person. 4. The risk for HIV infection can be reduced by having sex with an uninfected person. 5. The risk for HIV infection can be reduced by always using condoms for anal sex. 6. Showering or washing one's genital /private parts after sex prevents a person from getting HIV. 7. Using Vaseline or baby oil with condoms lowers the chance of getting HIV.

### Sexual behaviors

As shown in Table 2, 84.7% of the study population self-identified themselves as homosexual; only 20.7% of all MSM had never disclosed their sexual orientation. The median age at first sex with a male partner was 18 years [IQR: 17–20], without any significant difference between the two groups. The reported median number of male sexual partners over the past year was overall 3 [IQR: 1–10] and MSM willing to use the Internet to be informed on HIV were most likely to declare three or less male partners in last year. 10.3% of the respondents (21 MSM) had had sex with a woman in last year, without any difference between the two groups (Table 2).

### HIV risks

The assessment of HIV knowledge showed a relatively average level of HIV knowledge: 82 MSM (40.4%) had a score greater than five out of seven (Table 2). Almost 42% of the respondents considered themselves at no risk of HIV acquisition and a large majority, 189 MSM (93.1%), felt largely or sufficiently informed about HIV. These three factors were not significantly associated with a higher willingness to use the Internet to seek information on HIV prevention and care.

The rate of consistent condom use with a male partner in last month (always uses condoms when having sex) varied by type of sexual male partners, from 31.0% with a regular partner to 36.0% with a casual partner. Consistent lubricant use with a male partner in last month was reported by 37.4% of respondents. Condom or lubricant use was not significantly associated with the willingness to use the Internet to seek information on HIV prevention and care.

More than one quarter of the respondents (27.1%) reported having ever been screened or examined for STI. Lifelong HIV testing was reported by 43.3% of the MSM among which 68.2% (60) reported having undergone such a test and got the results. Overall, 42.4% of the MSM reported having ever been discriminated against in health services because of their sexual orientation. MSM willing to use the Internet to seek information on HIV prevention and care were most likely to have ever been screened or examined for STI, tested for HIV, to have got their HIV test results, and to have ever been discriminated in health services.

More than 18% of MSM (38) reported drinking alcohol more than once a week while 19.7% reported having ever used drugs (Table 2). The most popular drugs were ecstasy, methamphetamine and marijuana, respectively ever used by 26 MSM (11.7%), 25 MSM (11.3%), and 11 MSM (5%). Use of Poppers (6, 2.7%) and heroine (2, 0.9%) were more rarely reported.

Almost all respondents (99.1%) reported using the Internet and 73.4% reported having ever used it to seek male sexual partners, as shown in Table 2.

### Factors associated with the willingness to use the Internet to seek information on HIV prevention and care

In the univariable logistic regression analyses, Kinh ethnicity, less than or 3 partners in last year, history of HIV testing (with or without getting results), screening for STIs and seeking male sexual partners via Internet were shown to be significantly positively associated with the willingness to use the Internet to seek information on HIV prevention and care (Table 2). Furthermore, MSM who reported not having felt discriminated against in health services were more likely to report willingness to use the Internet to seek information on HIV prevention and care compared to MSM having experienced such a discrimination (Odds Ratio: 2.20, 95% Confidence Interval (CI) [1.15–4.34], Table 2).

In the multivariable regression model, factors significantly associated with the willingness to use the Internet to seek information on HIV prevention and care were a number of male partners in last year less than or equal to 3 (Adjusted Odds Ratio (aOR): 3.07, 95% CI [1.40–6.73]), a history of STI screening or examination (aOR: 4.10, 95% CI [1.02–16.48]), HIV testing (aOR: 3.23, 95% CI [1.20–8.64]) and having ever sought male sexual partner through Internet (adjusted OR: 3.56, 95% CI [1.55–8.18], Table 2).

## Discussion

Our main study result is the high willingness among MSM to use the Internet for HIV information purposes, with more than three quarters of the study respondents reporting their willingness to use the Internet to seek information on HIV prevention and care.

New media, such as Internet or mobile devices, may represent a potentially interesting channel to reach MSM in Vietnam. The challenge is now to identify which types of Internet tools are the most appropriate for addressing the HIV epidemic among them. Indeed, the scope of information on HIV prevention and care that can be provided is very wide, and several Internet-based strategies may be used, such as websites delivering basic information about HIV prevention and care or chat rooms and forums allowing visitors to ask questions [43]. Further than providing information, Internet could also contribute to referring users to MSM-friendly health care facilities, indicating for instance opening hours and medical services provided, HIV-test fees, or places where free condoms and lubricants are available. Whatever their intended uses, the development of these Internet tools will need to be adapted to MSM characteristics, needs and expectations. Within our study sample, 93.1% of MSM felt that they were sufficiently/well informed about HIV, meaning that new Internet tools aimed at increasing HIV prevention and care will need to be attractive and easy to use while also specific and precise. Qualitative research will be key to inform the definition of acceptable and targeted Internet tools for the Vietnamese heterogeneous MSM population. For example, by facilitating a tailored and personalized approach, Internet could be a way of bypassing the first clinic visit and initial face-to-face counseling, thus contributing to reduce stigma and discrimination preventing some MSM from accessing HIV prevention and care services [23], [44].

Another important study result was that the willingness to use the Internet to seek information on HIV prevention and care was associated with a previous history of HIV testing or STI screening, meaning that MSM with less access to health services may be those who are less likely to engage in online use for HIV prevention and care [45]. MSM who have been tested for HIV might gain some HIV information through health services. This experience of HIV services may have made these men more likely to be willing to get information on HIV in the future. HIV testing of MSM was shown to be instrumental in offering services tailored to their needs and accurate knowledge of HIV status is a key driver of prevention strategies adoption [46], [47]. However, we observe a strong majority of MSM unaware of their HIV status (70.4%), impacting on their willingness to engage in HIV prevention and care activities.

A positive association between the willingness to use the Internet to seek information on HIV prevention and care and having ever used the Internet for seeking sex partners was found. It should be underlined here that the proportion of urban MSM using the Internet to seek sex from male partners in our study (73.4%) is higher than that reported in 2007 by Colby *et al* (56%), likely due to higher rates of current use of the Internet. Because it is already commonly used by MSM in Vietnam, the Internet might readily become a tool for reaching MSM and improving their HIV knowledge. Furthermore, the expanding penetration of the Internet in Vietnam, from 3.1 million users in 2003 to 26.8 million users in 2010 is encouraging for the implementation of such use of Internet-based interventions for HIV prevention and care [35].

Finally, the observed association between being unwilling to use the Internet to seek information on HIV prevention and care and having more than three partners in last year shows that people with high-risk behaviors for HIV (multiple sex partners) are not aware of their risks and could be missed by prevention messages, even with the use of Internet.

This study has several limitations that need to be acknowledged. First, recruitment was based on a convenience sampling method. Thus our MSM sample may not be representative of all MSM in Ho Chi Minh City or in Vietnam, although high-risk behaviors reported have appeared to be similar as reported previously in Vietnam [10], [28], [48]. Second, potential bias may have derived from the choice of the MSM frequented venues and the fact that we were allowed to meet the MSM population only in three out of eight sites. Compared to the entire MSM population in the city, our sample might have been younger, better educated, and more likely to self-identify as homosexual. MSM groups wanting to hide their sexual orientation such as those who were married or had a high social status were probably less likely to be found in public locations frequented by gay men and therefore were less likely to be recruited at these sites. Third, although the questionnaires were self-administered, possible social desirability bias about sensitive questions should not be excluded, such as underreporting the number of male sexual partners or the frequency of unprotected sex or alcohol and drugs use. Furthermore, such a desirability bias could also partly explain the high rates of Internet use and willingness of the Internet use for HIV information purposes.

The high willingness to use the Internet to seek information on HIV prevention and care among MSM is an interesting result in the evolution of targeted tools for MSM in developing country settings. Although our study results are documented among a specific population group of young urban MSM affirming their homosexuality, they suggest directions for further research on Internet-based HIV prevention and care tools and interventions for a larger panel of MSM.

The recent increase of penetration of Internet in Vietnam, especially in rural areas, is a promising factor to consider developing such online programs to reduce the high-risk behaviors of MSM.

Future studies should first expand these results to a larger and more representative MSM population and then, in consultation with them, develop and test tailored online interventions to target the HIV prevention needs of Vietnamese MSM.
